# Association of Gonadotropin-Releasing Hormone Analogue Use With Subsequent Use of Gender-Affirming Hormones Among Transgender Adolescents

**DOI:** 10.1001/jamanetworkopen.2022.39758

**Published:** 2022-11-01

**Authors:** Andrea L. Nos, David A. Klein, Terry A. Adirim, Natasha A. Schvey, Elizabeth Hisle-Gorman, Apryl Susi, Christina M. Roberts

**Affiliations:** 1 Division of Adolescent Medicine, Children’s Mercy Kansas City, Kansas City, Missouri; 2 Department of Pediatrics, University of Missouri-Kansas City School of Medicine, Kansas City; 3 Department of Family Medicine, Uniformed Services University, Bethesda, Maryland; 4 Department of Pediatrics, Uniformed Services University, Bethesda, Maryland; 5 Department of Family Medicine, David Grant Medical Center, Travis Air Force Base, California; 6 Department of Preventive Medicine and Biostatistics, Uniformed Services University, Bethesda, Maryland; 7 Department of Medical and Clinical Psychology, Uniformed Services University, Bethesda, Maryland; 8 Department of Preventive Medicine and Biostatistics, Uniformed Services University, Bethesda, Maryland

## Abstract

**Importance:**

Gonadotropin-releasing hormone analogue (GnRHa) use during puberty improves mental health among transgender and gender-diverse (TGD) adolescents. In previous studies, most (96.5%-98.1%) TGD adolescents who started GnRHa subsequently started gender-affirming hormones (GAH), raising concerns that GnRHa use promotes later use of GAH.

**Objective:**

To determine whether GnRHa use among TGD adolescents is associated with increased subsequent GAH use.

**Design, Setting, and Participants:**

This is a retrospective cohort study of administrative records collected between 2009 and 2018. The current analysis was completed in August 2022. Participants were enrolled in the US Military Healthcare System (MHS) with an initial TGD-related encounter occurring between ages 10 and 17 years.

**Exposures:**

GnRHa use.

**Main Outcomes and Measures:**

Initiation of GAH.

**Results:**

The 434 patients were a mean (SD) of 15.4 (1.6) years old at the time of their first TGD-related encounter; 312 (71.9%) were assigned female at birth, and 300 (69.1%) had an enlisted insurance sponsor. GnRHa use was more common among patients who were assigned male at birth (28 patients [23.0%]) than those assigned female (42 patients [13.5%]), but GAH use was not. Socioeconomic status was not associated with GnRHa or GAH use. Compared with older patients (aged 14-17 years), those who were younger (aged 10-13 years) at the time of the initial TGD-related encounter had a higher rate of GnRHa use (32 patients [57.1%] vs 38 patients [10.1%]) and a longer median time to starting GAH. The median interval from the date of the initial encounter to starting GAH decreased over time, from 2.3 years (95% CI, 1.7-2.8 years) between October 2009 and December 2014 to 0.6 years (95% CI, 0.5-0.6 years) between September 2016 and April 2018. Patients who were prescribed GnRHa had a longer median time to starting GAH (1.8 years; 95% CI, 1.1-2.4 years) than patients who were not (1.0 years; 95% CI, 0.8-1.2 years) and were less likely to start GAH during the 6 years after their first TGD-related encounter (hazard ratio, 0.52; 95% CI, 0.37-0.71). Among 54 younger (aged 10-13 years) patients who were not eligible to start GAH at their first encounter, GnRHa use was associated with a longer median time to starting GAH, but age at the first TGD-related visit was not.

**Conclusions and Relevance:**

In this cohort study of TGD adolescents, GnRHa use was not associated with increased subsequent GAH use. These findings suggest that clinicians can offer the benefits of GnRHa treatment without concern for increasing rates of future GAH use.

## Key Points

**Question:**

Is there an association between use of gonadotropin-releasing hormone analogue and subsequent use of gender-affirming hormones among transgender and gender-diverse adolescents?

**Findings:**

In this cohort study of 434 adolescents, there was no significant association between gonadotropin-releasing hormone use and subsequent initiation of gender-affirming hormones.

**Meaning:**

These findings suggest that clinicians can offer gonadotropin-releasing hormone analogues to transgender and gender-diverse adolescents during pubertal development for mental health and cosmetic benefits without an increased likelihood of subsequent use of gender-affirming hormones.

## Teaser

This cohort study examines whether use of gonadotropin-releasing hormone analogue (GnRHa) among transgender and gender-diverse adolescents is associated with subsequent use of gender-affirming hormones.

## Introduction

In the US, 1.8% of high school students identify as transgender, with an increasing number of children and adolescents seeking gender identity–specific health care in recent years.^1,2,3,4^ Transgender and gender-diverse (TGD) youth are at high risk for depression, anxiety, self-harm, and suicidality.^1,5,6,7^ This is likely related to gender dysphoria, family rejection, and increased exposure to bullying, discrimination, harassment, violence, and social isolation.^1,6^

Multiple medical societies and evidence-based treatment guidelines support the use of gonadotropin-releasing hormone analogues (GnRHa) to reversibly suppress further pubertal development in peripubertal youth with gender dysphoria.^8,9,10,11^ This treatment is associated with improvements in global functioning, depression, suicidal ideation, and overall behavioral and emotional problems among youth with gender dysphoria.^12,13,14,15,16^ Treatment with GnRHa can improve gender dysphoria by reversibly halting development of secondary sex characteristics that are not consistent with the patient’s experienced gender. Relieving the pressure associated with additional pubertal development can also give young adolescents more time to fully confirm their gender identities before making a decision about initiating further gender-affirming treatment with irreversible effects.^8,9,17^ Pubertal suppression may also affect cosmetic outcomes if the patient elects to progress to gender-affirming hormones, decreasing the need for certain future interventions, such as mastectomies in people with trans-masculine gender identities and facial feminization in people with trans-feminine gender identities.^18^ However, the use of GnRHa for pubertal suppression is associated with short-term physical symptoms, such as headaches, hot flashes, and fatigue, and long-term risks such as decreased bone mineral density and changes in body composition. Use of GnRHa in early puberty followed by treatment with estrogen can also impair fertility and complicate future vaginoplasty.^19,20,21,22^ The benefits of GnRHa treatment in postpubertal youth are not as clear. Assisting patients and families with assessment of the risks and benefits of treatment and managing adverse effects that occur is an important part of caring for transgender youth.

In prior studies^4,20,23^ of gender-affirming medical care for TGD adolescents, 96.5% to 98.1% of individuals who started GnRHa subsequently used gender-affirming hormones. This high rate has led some clinicians, judges, and legislators to express concerns that initiation of GnRHa treatment in young adolescents does not serve as a pause in pubertal development, but instead inappropriately advances the decision to start gender-affirming hormones to a younger age when the adolescent has not yet completed the cognitive development required to assent to this treatment.^8,24,25,26,27^ Guidelines for gender-affirming care suggest that most adolescents do not reach cognitive maturity until age 15 to 16 years, after most have completed puberty and can no longer obtain maximum benefit from GnRHa treatment.^8^

In the United Kingdom, a court ruled that GnRHa treatment could not be administered to transgender patients younger than 16 years without a court order because they assert that this treatment inevitably leads to use of gender-affirming hormones. This ruling also suggested that gender-affirming care for patients aged 16 and 17 years old should be restricted as well.^26^ In the US, 3 states have outlawed all gender-affirming medical care for minors, 1 state government has taken administrative action to classify gender-affirming medical care for minors as child abuse, and 16 state public medical insurance programs for those with limited income or resources (Medicaid) do not pay for gender-affirming medical care. An additional 19 state legislatures are considering laws to make some or all aspects of gender-affirming medical care for minors illegal.^28,29^

It is unknown whether GnRHa treatment among TGD youth leads to an increase in subsequent use of gender-affirming hormones or whether the high continuation rate seen in previous studies reflects the natural history of gender dysphoria among adolescents or the rigorous screening process used before administration of GnRHa in previous studies. The purpose of our study is to compare the rates of gender-affirming hormone initiation between patients who are treated with GnRHa and those who are not treated with GnRHa. We hypothesized that TGD youth who were treated with GnRHa would not have a higher rate of future gender-affirming hormone use compared with TGD youth who were not treated with GnRHa.

## Methods

This is a retrospective cohort study examining the association between GnRHa use and subsequent use of gender-affirming hormones among TGD youth enrolled in the US Military Health System (MHS) between October 2009 and April 2018 and using their TRICARE health plan benefit. TRICARE Prime is the medical health plan benefit for almost 4.8 million people stationed around the world, including active-duty service members in the US Army, Air Force, Marines, and Navy; military retirees; and their families. Data for this study were extracted from the Military Health System Data Repository (MDR) by a member of the research team (A.S.) in 2019. The MDR includes insurance billing records for inpatient care, outpatient care, and outpatient prescriptions that were paid for by TRICARE. This study was approved by the authors’ local institutional review boards as a secondary analysis of deidentified, preexisting data. Consent was not needed because the data were deidentified, in accordance with 45 CFR, subpart A, §46.104, exempt research, exception 4. This manuscript was prepared in accordance with the Reporting of Studies Conducted Using Observational Routinely-Collected Health Data (RECORD) statement.

In a previous study,^7^ using the same data source as this study, the investigators identified 3754 youth, younger than 18 years, who had at least 1 health care encounter with a transgender-related diagnosis. For this study, we further refined our sample population using the inclusion criteria outlined here. Patients were required to have at least 2 distinct encounters associated with 1 or more of the following codes: *International Classification of Diseases, Ninth Revision *codes 302.6, 302.85, 302.50, 302.51, 302.52, and 302.53; and *International Statistical Classification of Diseases and Related Health Problems, Tenth Revision *codes F64.0, F64.1, F64.2, F64.8, and Z87.890. The initial TGD-related visit had to occur between ages 10 and 17 years, and the patient needed to have at least 1 medical encounter, for any reason, occurring after their 14th birthday. On the basis of current guidelines, patients are required to have the cognitive capacity to provide meaningful informed consent or assent to treatments with permanent effects before starting gender-affirming hormones. Some adolescents have developed this level of cognitive capacity as early as age 14 years, and most have developed it by age 16 years.^8^ Therefore, age 14 years is generally the earliest a clinician will consider prescribing a patient gender-affirming hormones. Requiring a patient to have at least 1 clinical encounter after turning 14 years old provided all subjects an opportunity to start hormone therapy during our study period.

Pharmacy records were accessed to identify all prescriptions for GnRHa and initial prescriptions for gender-affirming hormones occurring between 30 days before their first clinic visit addressing gender dysphoria and 90 days after their final clinic visit addressing gender dysphoria. Discontinuation of GnRHa treatment was defined as no GnRHa prescriptions for a period 3 times as long as their most recent prescription length. Initiation of gender-affirming hormones was defined as first use of sex hormones inconsistent with their sex assigned at birth. We used sex indicated by the earliest gender marker recorded in the MDR as a proxy for sex assigned at birth. We excluded patients if their first prescription for gender-affirming hormones occurred before or at the same time as starting GnRHa.

Demand for gender-affirming medical care for pediatric age patients increased during our study period. Demand peaked in 2016 when TRICARE officially authorized payment for gender-affirming medical care, on September 1, 2016.^30,31^ We created a measure of time by dividing our sample of patients into 3 roughly equal-sized groups according to the date of their initial TGD-related encounter. Our periods included the period of lower demand for gender-affirming care before official approval of payment for gender-affirming care by TRICARE (October 2009 to December 2014), the period of increased demand for gender-affirming care before official authorization of care (January 2015 to August 2016), and the period after insurance approval (September 2016 to April 2018). We also collected insurance sponsor’s rank (officer vs enlisted) as a proxy for socioeconomic status on the basis of the assumption that an enlisted insurance sponsor would have a lower income than an officer while on active duty, and most officers have a college education whereas many enlisted sponsors do not.^32^

### Statistical Analysis

The analysis for this study was completed in August 2022. We used Kaplan-Meier survival analyses to estimate the time from the patient’s first encounter for a TGD-related diagnosis to initiation of gender-affirming hormones. We also assessed the association of time to initiation of gender-affirming hormones with GnRHa use and demographic factors. Patients were censored if they reached their final billable clinical encounter in the MHS before the end of the study period. We used the Breslow (generalized Wilcoxon) χ^2^ test to assess the bivariate association of demographic factors, TRICARE transgender coverage status (officially approved vs unapproved), and date of initial transgender-related encounter with time to starting hormones. We used bivariable and multivariable Cox proportional hazard analyses to assess the independent association of our variables on the hazard of starting gender-affirming hormones. We used visual inspection of the log-log survival curve to ensure that the proportional hazard assumption was not violated. Our threshold for statistical significance in this study was *P *< .05 for 2-sided tests.

We conducted a subanalysis of the association between GnRHa use and time to initiation of gender-affirming hormones among patients who were aged 10 to 13 years at the time of their initial TGD-related medical encounter. Previous research has suggested that age 10 to 13 years is a key period in the development of gender identity and persistence or desistence of gender dysphoria.^33^ Many patients are also experiencing pubertal development during this period and would obtain the most benefit from GnRHa treatment. SPSS statistical software version 28.0.1.0 (IBM) was used for statistical analysis.

## Results

We identified 434 TGD youth who met our inclusion criteria: 143 with an initial encounter in October 2009 to December 2014, 133 in January 2015 to August 2016, and 158 in September 2016 to April 2018. Most patients were assigned female at birth (312 patients [71.9%]) and had an enlisted insurance sponsor (300 patients [69.1%]). The mean (SD) age at the first TGD-related encounter was 15.4 (1.6) years. The number of patients presenting for gender-affirming medical care increased over time. The mean age at the first TGD-related encounter and the ratio of those assigned male to female did not change over time (Table 1).

**Table 1.** Transgender Individuals Aged 10 to 17 Years Seeking Gender-Affirming Medical Care in the Military Heath System by Year of First Transgender-Related Encounter, 2009-2018

Characteristic	Participants, No. (%)			
	TRICARE did not officially cover gender-affirming medical care		TRICARE officially covered gender-affirming medical care, September 2016 to April 2018 (n = 158)^c^	Total sample, October 2009 to April 2018 (N = 434)
	October 2009 to December 2014 (n = 143)^a^	January 2015 to August 2016 (n = 133)^b^		
Age at first transgender-related encounter, mean (SD), y	15.2 (1.8)	15.4 (1.6)	15.6 (1.2)	15.4 (1.6)
Age group at first transgender-related encounter, y				
10-13	22 (15.4)	22 (16.5)	12 (7.4)	56 (12.9)
14-17	121 (84.6)	111 (83.5)	146 (92.6)	378 (87.1)
Sex assigned at birth				
Male	47 (32.9)	35 (26.3)	40 (25.2)	122 (28.1)
Female	96 (67.1)	98 (73.7)	118 (74.8)	312 (71.9)
Parent enlisted or an officer^d^				
Enlisted	99 (69.2)	93 (69.9)	108 (68.1)	300 (69.1)
Officer	44 (30.8)	40 (30.1)	50 (31.9)	134 (30.9)
Prescribed gonadotropin-releasing hormone analogue	19 (13.3)	27 (20.0)	24 (15.2)	70 (16.1)

^a^ There were 20 new patients in October 2009 to December 2010, 15 new patients in 2011, 20 new patients in 2012, 34 new patients in 2013, and 54 new patients in 2014.

^b^ There were 92 new patients in 2015 and 41 new patients in 2016.

^c^ There were 64 new patients September 2016 to December 2016 and 94 new patients January 2017 to April 2018.

^d^ Denotes current rank or rank before retirement from the military.

The median interval between the first TGD-related encounter and starting gender-affirming hormones was 1.1 years (95% CI, 0.9-1.3 years). Within 1 year of the initial TGD-related encounter, an estimated 46.4% of TGD adolescents aged 10 to 17 years (95% CI, 41.7%-51.1%) had started gender-affirming hormones. Within 4 years of the initial encounter, 88.3% of TGD adolescents (95% CI, 84.8%-91.8%) had started gender-affirming hormones.

The median interval between the first TGD-related encounter and starting hormones decreased over time, from 2.3 years (95% CI, 1.7-2.8 years) between October 2009 and December 2014 to 0.6 years (95% CI, 0.5-0.6 years) between September 2016 and April 2018. Hormone use at 18 months after diagnosis increased with recency of first diagnosis date: 36.6% of adolescents (95% CI, 28.8%-44.4%) in October 2009 to December 2014, 65.4% (95% CI, 58.0%-72.8%) in January 2015 to August 2016, and 95.3% (95% CI, 87.3%-100%) in September 2016 to April 2018. TGD youth who were initially seen after September 2016 had an almost 5 times higher hazard of starting gender-affirming hormones (hazard ratio, 4.92; 95% CI, 3.59-6.74) than patients who had an initial transgender-related encounter between 2009 and 2014.

**Figure 1.**
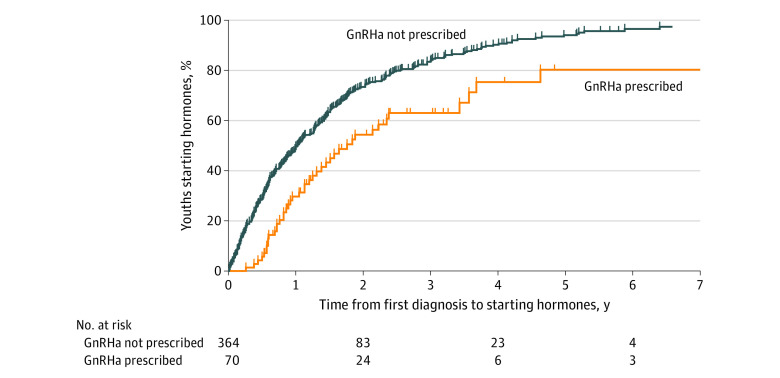
Use of Gonadotropin-Releasing Hormone Analogues (GnRHa) and Gender-Affirming Hormones Among Transgender Youth Aged 10 to 17 Years

**Figure 2.**
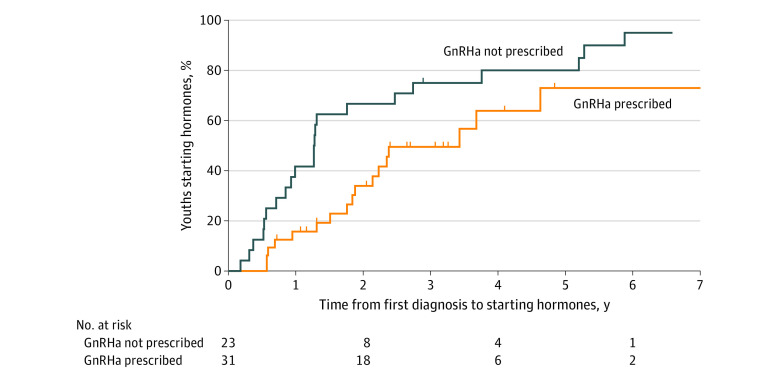
Use of Gonadotropin-Releasing Hormone Analogues (GnRHa) and Gender-Affirming Hormones Among Transgender Youth Aged 10 to 13 Years

Patients who were 10 to 13 years old at the initial TGD-related encounter were more likely than patients who were 14 to 17 years old to be prescribed GnRHa (32 patients [57.1%] vs 38 patients [10.1%]) and had a larger interval between the initial TGD-related appointment and starting gender-affirming hormones (Tables 2 and 3). Compared with patients without GnRHa use, GnRHa use was associated with a longer median gap between the initial appointment and starting gender-affirming hormones (1.8 years [95% CI, 1.1-2.4 years] vs 1.0 years [95% CI, 0.8-1.2 years]) and a lower hazard of starting gender-affirming hormones (hazard ratio, 0.52; 95% CI, 0.37-0.71) (Figure 1). This association was consistent across time periods. We saw this same association when we restricted our analyses to 54 patients who were aged 10 to 13 years at the time of their first TGD-related encounter (Figure 2). Most patients (63 of 70 patients [90%]) who started GnRHa continued treatment until they started gender-affirming hormones or were censored from further analysis. Of the 7 patients in our study who started and then stopped GnRHa treatment while continuing to seek medical care using their TRICARE benefit, 3 went on to start testosterone.

**Table 2.** Gonadotropin-Releasing Hormone Analogue Use

Demographics	Patients started using a gonadotropin-releasing hormone analogue, No. (%) (N = 434)	
	No (n = 364)	Yes (n = 70)
Sex assigned at birth^a^		
Male	94 (77.0)	28 (23.0)
Female	270 (86.5)	42 (13.5)
Age at first transgender diagnosis, y^a^		
10-13	24 (42.9)	32 (57.1)
14-17	340 (88.9)	38 (10.1)
Insurance sponsor rank		
Enlisted	251 (83.7)	49 (16.3)
Officer	113 (84.3)	21 (15.7)

^a^ Differences between groups were statistically significant at *P* < .05.

**Table 3.** Association of Patient Factors With Initiation of Gender-Affirming Hormones

Factor	Time from initial encounter to initiation of gender-affirming hormones (KM estimate), median (95% CI), y	HR (95% CI)^a^	
		Hazard of starting hormones (bivariable analyses)	Independent hazard of starting hormones (multivariable analyses)
Total sample	1.1 (0.9-1.3)	NA	NA
Date of initial diagnosis			
October 2009 to December 2014	2.3 (1.7-2.8)	1 [Reference]	1 [Reference]
January 2015 to August 2016	1.1 (0.9-1.3)	2.25 (1.71-2.97)	2.43 (1.84-3.21)
September 2016 to April 2018^b^	0.6 (0.5-0.6)	4.92 (3.59-6.74)	5.12 (3.72-7.04)
Prescribed gonadotropin-releasing hormone analogue			
No	1.0 (0.8-1.2)	1 [Reference]	1 [Reference]
Yes	1.8 (1.1-2.4)	0.52 (0.37-0.71)	0.54 (0.38-0.77)
Age at initial diagnosis, y			
10-13	2.1 (1.5-2.8)	1 [Reference]	1 [Reference]
14-17	1.0 (0.9-1.2)	2.03 (1.46-2.84)	1.42 (0.98-2.07
Sponsor’s rank^c^			
Enlisted	1.1 (0.9-1.3)	1 [Reference]	NA^d^
Officer	1.0 (0.7-1.3)	1.06 (0.85-1.33)	
Sex assigned at birth			
Male	1.3 (1.0-1.5)	1 [Reference]	NA^d^
Female	1.1 (0.9-1.2)	1.25 (0.99-1.58)	

Abbreviations: HR, hazard ratio; KM, Kaplan-Meier; NA, not applicable.

^a^ HRs and 95% CIs were determined by Cox proportional hazards analyses.

^b^ On September 1, 2016, TRICARE adjusted the list of covered conditions to include gender-affirming medical rendered to military retirees and family members of active duty and retired service members.

^c^ Enlisted service members are required to complete high school, whereas officers are required to complete college before joining the US military. Officers also have higher pay while in the military and military pension after retirement. We are using this factor as a proxy for family income.

^d^ Not included in multivariable model.

GnRHa use was more common among patients who were assigned male at birth than those who were assigned female (28 patients [23.0%] vs 42 patients [13.5%]) but GAH use was not (Table 3). Sponsor’s military rank (a proxy for socioeconomic status) was not associated with initiation of GnRHa or gender-affirming hormones (Table 3). In multivariable analysis including GnRHa use, time of the initial transgender-related encounter, and patient age at the time of the first encounter, only GnRHa use and time of the initial encounter had an independent association with the hazard of initiating gender-affirming hormones.

## Discussion

The findings of this cohort study suggest that GnRHa treatment is not associated with increased progression to gender-affirming hormones among treatment-seeking TGD adolescents in a population receiving low or no-cost medical care in the MHS. We also found that number of patients per year presenting for gender-affirming medical care increased during our study period, whereas the time between the initial TGD-related encounter and initiation of gender-affirming hormones decreased, but the median age at presentation remained the same. Patients who were older at the time of the initial TGD-related encounter had a shorter median time between the first medical encounter and initiation of gender-affirming hormones and were more likely to start gender-affirming hormones in general. We also found that assigned male patients were significantly more likely than assigned female patients to start GnRHa, but not gender-affirming hormones. In a medical system with low or no-cost medical care, the sponsor’s military rank (a proxy for socioeconomic status) was not associated with differences in use of gender-affirming hormones by TGD adolescents.

Across the MHS, we saw lower rates of progression from GnRHa treatment to gender-affirming hormones among TGD adolescents than were seen in studies of specialized gender clinics in Europe (92%-98%).^4,20,23^ Some of the national centers in Europe examined in prior studies had more stringent criteria for initiation of gender-affirming treatment than patients seen in the MHS, such as requiring patients to have at least 6 months of involvement with child psychology or psychiatry before starting GnRHa treatment,^4,21^ requiring patients to have persistent gender dysphoria since childhood that increased with puberty,^13,17,21^ or requiring patients to start GnRHa treatment before obtaining access to gender-affirming hormones.^23^ These additional criteria may influence patterns of GnRHa use and account for some of the difference observed in progression from GnRHa use to gender-affirming hormone use. We also included younger children in our study compared with previous studies, and clinicians in the MHS initiated GnRHa at younger ages than in previous studies.^4,5^ In our study, younger patients were less likely to initiate gender-affirming hormones; therefore, inclusion of these patients might also account for the lower rate of gender-affirming hormone use seen in our study. This lower rate of starting gender-affirming hormone therapy among younger patients is consistent with previous studies finding that age 10 to 13 years is a key period for confirmation of gender identity and determining whether gender dysphoria will persist or resolve.^33^ TGD patients first presenting at an older age may have passed through this period of identity consolidation and are more likely to have persistent gender dysphoria. However, consistent with other studies, few TGD patients discontinued treatment.

Use of GnRHa treatment in younger adolescents is reversible and associated with improvements in mental health and cosmetic outcomes if gender dysphoria persists.^8,9,12,13,14,15,16,17,18^ In our study, we found that use of GnRHa treatment among adolescents did not collectively increase future use of gender-affirming hormones. This suggests that clinicians can offer GnRHa treatment to young TGD adolescents without an increased likelihood of future use of gender-affirming hormones.

### Limitations

This study has limitations that should be addressed. It is a retrospective cohort analysis of administrative data from patients enrolled in the US military health plan program, TRICARE. The children of active duty or retired service members identified in our study are different from the general population in several ways. Compared with the general population in the US, children of active duty and retired service members have families with a higher average socioeconomic standing, higher levels of parental education, medical care coverage with lower out-of-pocket expenses and more comprehensive health care coverage, and higher geographic mobility. These factors limit the generalizability of our findings. As this is a study of health plan administrative data, we do not have information on care obtained by the family without using their TRICARE benefit. In the US, medical expenses can be covered by employer-funded private medical insurance, government insurance programs for the economically disadvantaged and elderly adults, individually purchased private insurance obtained with or without cost subsidies from the government, and paying out of pocket at the time of service. Individuals can have multiple types of insurance at the same time, such as a military retiree with TRICARE and commercial insurance through their employer, and can select which program they use to pay for health care services. It is possible that patients elected to obtain gender-affirming medications without using their TRICARE benefit, while still obtaining other medical care in the MHS. However, the difference in cost between commercial insurance programs and TRICARE make this less likely. The average out-of-pocket medication costs would be $500 per year using commercial insurance vs $0 to $36 per year using TRICARE for a transgender woman and $230 per year vs $0 to $72 per year for a transgender man.^34^ We also did not capture information on individual patient, parent, and clinician factors that may influence decisions about starting or stopping gender-affirming medical treatments, or to seek out these treatments at all. Future studies examining the individual patient, family, clinician, and governmental factors influencing initiation and discontinuation of gender-affirming medications, conducted with more generalizable samples, would be helpful to clinicians when counseling patients and families on the risks and benefits of gender-affirming medications and monitoring treatment.

## Conclusions

In this cohort study of transgender adolescents, GnRHa use was not associated with an increased hazard of subsequent gender-affirming hormone use. These data suggest that clinicians can offer the benefits of GnRHa treatment to TGD youth with gender dysphoria without concern for unduly or inappropriately increasing rates of subsequent gender-affirming hormone use.
